# Pathomorphological Patterns of Placental Dysfunction in Late Manifestation of Fetal Growth Restriction

**DOI:** 10.15388/Amed.2025.32.2.21

**Published:** 2025-12-30

**Authors:** Oksana Zhurakivska, Yuliia Yarotska, Dmytro Govsieiev, Oksana Ostrovska, Mariana Rymarchuk

**Affiliations:** 1 Department of Human Anatomy, Ivano-Frankivsk National Medical University of the Ministry of Health of Ukraine, Ivano-Frankivsk, Ukraine; 2 Obstetrics and Gynecology Department, Perinatal Center of Kyiv, Kyiv, Ukraine; 3 Department of Obstetrics and Gynecology No. 1, Bogomolets National Medical University, Kyiv, Ukraine; 4 Department of Obstetrics and Gynecology of Postgraduate Education, Ivano-Frankivsk National Medical University of the Ministry of Health of Ukraine, Ivano-Frankivsk, Ukraine; 5 Department of Obstetrics and Gynecology of Postgraduate Education, Ivano-Frankivsk National Medical University of the Ministry of Health of Ukraine, Ivano-Frankivsk, Ukraine

**Keywords:** fetal growth restriction, placenta, maternal vascular malperfusion, fetal vascular malperfusion, postnatal macro-histomorphological examination, vaisiaus augimo sulėtėjimas, placenta, motinos kraujagyslių kraujotakos sutrikimas, vaisiaus kraujagyslių kraujotakos sutrikimas, postnatalinis makrohistomorfologinis tyrimas

## Abstract

Fetal growth restriction is one of the main factors contributing to neonatal mortality, stillbirth, and cardiovascular dysfunction in a fetus and newborn, where one of the common causes is placental dysfunction; it can occur as a result of placental vascular anomalies, which requires clarification and systematization of existing scientific provisions based on the assessment of morphological patterns of fetoplacental circulatory disorders.

The aim of the study was to investigate and evaluate morphological markers of fetoplacental blood flow disorders in late manifestation of fetal growth restriction by pathohistological examination of placental tissue.

**Materials and methods:**

Clinical and anamnestic features were assessed in 80 pregnant women with late manifestation of fetal growth restriction (the main group of research) and in 40 patients with a timely labour and birth of fetuses with normal fetometry parameters for the gestational age. 32 and 10 placental tissue samples were selected in the main group and in the comparison group, respectively, for pathohistological examination, and a postnatal macro-histomorphological assessment of the placenta was performed.

**Results:**

Postnatal macromorphometric characteristics of placental tissue reflect pathological features of its formation, dominated by an abnormal shape (46–57.5%), eccentric umbilical cord insertion (43–53.8%) with main and intermediate types of vascular branching (37–46.2%), and a statistically lower placental weight and diameter. A combination of maternal and fetal vascular malperfusion was noted, with the following most significant markers: infarctions (13–40.6%), distal villous hypoplasia (24–75.0%), increased syncytial nodules as a manifestation of delayed villous maturation as a manifestation of premature maturation (24–75.0%), decidual arteriopathy (13–40.6%). Analysis of histopathological data indicates malperfusion of the fetal vessels, with the proportion of villitis and markers of intrauterine infection verified in one third of the samples (13–40.6%).

**Conclusions:**

In cases of late-onset fetal growth restriction, placental lesions occur with the development of maternal and fetal vascular malperfusion. Pathomorphological criteria of maternal vascular malperfusion are statistically significant in the main group: infarctions, distal villous hypoplasia, decidual arteriopathy, and chorionic villous dysmaturation. Fetal vascular malperfusion is characterized by obliteration and thrombosis of the stem vessels of the anchoring and intermediate villi, avascularization, hyalinosis of the villi, and, less frequently, delayed maturation with the development of stromal-vascular karyorexis. In our study, both Type 1 and Type 2 fetal vascular malperfusion in different placentas with fetal growth restriction were found, but the segmental type was still the most common.

## Abstract (it)

**Įvadas:**

Vaisiaus augimo sulėtėjimas yra vienas iš pagrindinių veiksnių, lemiančių naujagimių mirtingumą, negyvagimius bei vaisiaus ir naujagimio širdies ir kraujagyslių funkcijos sutrikimus. Viena iš dažniausių priežasčių yra placentos funkcijos sutrikimas; jis gali atsirasti dėl placentos kraujagyslių anomalijų, todėl reikia išsiaiškinti ir sisteminti esamas mokslo žinias, remiantis vaisiaus ir placentos kraujotakos sutrikimų morfologinių modelių vertinimu. Tyrimo tikslas buvo ištirti ir įvertinti morfologinius vaisiaus ir placentos kraujotakos sutrikimų žymenis vėlyvuoju vaisiaus augimo sulėtėjimo laikotarpiu, atliekant placentos audinio histologinį tyrimą.

**Medžiagos ir metodai:**

Klinikiniai ir anamnezės požymiai buvo vertinami 80 nėščių moterų, kurioms vėlai pasireiškė vaisiaus augimo sulėtėjimas (pagrindinė grupė), ir 40 pacienčių, kurios gimdė laiku ir kurių vaisius turėjo normalius vaisiaus matmenų parametrus, atitinkančius nėštumo savaites. Pagrindinės grupės ir lyginamosios grupės pacienčių buvo atrinkti atitinkamai 32 placentos audinių mėginiai ir 10 placentos audinių mėginių histologiniam tyrimui, taip pat atliktas placentos postnatalinis makrohistomorfologinis vertinimas.

**Rezultatai:**

Postnatalinės makromorfometrinės placentos audinio charakteristikos rodo jo formavimosi patologines savybes, kurioms būdinga nenormali forma (46–57,5 %), išorinė virkštelės prisitvirtinimo vieta (43–53,8 %) su pagrindiniais ir tarpiniais kraujagyslių šakojimosi tipais (37–46,2 %) bei statistiškai mažesnis placentos svoris ir skersmuo. Buvo pastebėta motinos ir vaisiaus kraujagyslių kraujotakos sutrikimų kombinacija, kurios svarbiausi žymenys buvo šie: infarktas (13–40,6 %), distalinė choriono gaurelių hipoplazija (24–75,0 %), padidėję sincitiniai mazgeliai dėl choriono gaurelių brendimo vėlavimo kaip priešlaikinio brendimo pasireiškimas (24–75,0 %), decidualinė arteriopatija (13–40,6 %). Histopatologinių duomenų analizė rodo vaisiaus kraujagyslių kraujotakos sutrikimą, viliozės ir intrauterinės infekcijos požymių proporcija patvirtinta trečdalyje mėginių (13–40,6 %).

**Išvados:**

Vėlyvojo vaisiaus augimo sulėtėjimo atveju placentos pažeidimų atsiranda kartu su motinos ir vaisiaus kraujagyslių kraujotakos sutrikimu. Motinos kraujagyslių kraujotakos sutrikimo patologiniai kriterijai yra statistiškai reikšmingi pagrindinėje grupėje: infarktas, distalinė gaurelių hipoplazija, decidualinė arteriopatija ir choriono gaurelių nesubrendimas. Vaisiaus kraujagyslių nepakankama kraujotaka pasireiškia tvirtinamųjų ir tarpinės viliozės stiebo kraujagyslių obliteracija ir tromboze, avaskuliarizacija, choriono gaurelių hialinoze ir rečiau vėluojančiu brendimu su stromos kraujagyslių karioreksijos išsivystymu. Mūsų tyrime buvo nustatyta tiek 1 tipo, tiek 2 tipo vaisiaus kraujotakos sutrikimų skirtingose placentose, kai vaisiaus augimas sulėtėjęs, tačiau segmentinis tipas buvo labiausiai paplitęs.

## Introduction

Worldwide, *Fetal Growth Restriction* (FGR) is one of the main factors contributing to neonatal mortality, stillbirth and cardiovascular dysfunction in fetuses and newborns [1, 2]. The scientific community considers FGR to be a polyetiological universal condition caused by a deficiency of oxygen, macro- and micronutrients, and a decrease in metabolic processes against the background of pathological placental perfusion [3–5]. Complicating about 10.0% of all pregnancies, FGR currently causes significant fluctuations in the structure of perinatal morbidity and mortality, increases the risk of antenatal fetal death, and creates a negative background for the future development of the newborn [3–4].There are many possible causes of FGR, but it is believed that most cases not associated with congenital fetal malformations, genetic abnormalities or infectious etiology are caused by impaired uteroplacental blood flow and the development of placental dysfunction [6]. At birth, the fetoplacental weight ratio provides a retrospective view of the placenta’s ability and effectiveness in supporting fetal growth, and also creates the prerequisites for assessing the potential risks of chronic conditions and diseases in a newborn in the future through developmental programming. The physiological course of pregnancy is ensured by complex mechanisms of adaptive processes in the mother-placenta-fetus system, where the formation of an additional vascular pool (uteroplacental blood flow) in a pregnant woman is important [7]. Adequate uteroplacental blood flow, along with satisfactory villous angiogenesis, are key factors necessary for the full development and functioning of the placenta, as well as further fetal growth [6].

One of the common causes of FGR is placental dysfunction, which can occur as a result of placental vascular abnormalities [8–9]. Interest in studying the histological patterns of placental dysfunction has increased with the accumulation of scientific data on the multifunctionality of placental tissue and the development of hemodynamic disorders, neuroimmune interactions and non-infectious inflammatory alterations, failure of adaptive functions, prolonged prenatal stress, chronic hypoxia, and metabolic deficiency. Currently, the literature does not fully and consistently describe the structural changes in the vascular bed of this complex system, and morphological criteria have not been developed that would allow pathologists to determine the severity of maternal malperfusion, which, in turn, would direct the scientific search of clinicians and practitioners towards the identification of adequate preventive measures capable of influencing the correction of hypoxic-hypodynamic disorders in such newborns [10].

The analysis of literature reports requires clarification and systematization of existing scientific provisions and creates prerequisites for the development of practical recommendations based on the assessment of morphological patterns of fetoplacental blood flow disorders, as well as the use of accumulated knowledge in clinical practice.

**The aim of the study** was to investigate and evaluate morphological markers of fetoplacental blood flow disorders in late manifestation of fetal growth restriction by means of pathohistological examination of placental tissue.

## Materials and methods

This work is part of the research conducted by the Department of Obstetrics and Gynecology No. 1 of the Bogomolets National Medical University, entitled “*Preservation and Restoration of Women’s Reproductive Health in Conditions of Rapid Social and Medical Change*” (State Registration No. 0123U100920), in which one of the authors is a co-implementing member of the topic. The scientific research was conducted in accordance with the principles of the Helsinki Declaration on Biometric Research and the GCH ICH (1996), the Council of Europe Convention on Human Rights, as well as the relevant Ukrainian laws on experimental and clinical research, in accordance with biometric standards and in compliance with the principles of confidentiality and medical ethics.

During the course of the scientific research, an assessment of clinical and anamnestic characteristics was carried out in 80 pregnant women whose gestation was complicated by late manifestation of fetal growth restriction (the main group) and in 40 patients with timely delivery and birth of fetuses with normal fetometry parameters for the gestational age. International criteria used for the diagnosis of FGR were: birth weight <3 percentile or a combination of three criteria: birth weight <10 percentile; head circumference <10 percentile; prenatal diagnosis of FGR and prenatal risk factors associated with FGR [2].

The diagnosis of late FGR was based on the Delphi method [11] and was established at 32 weeks or later, taking into account the criteria defined by this procedure: estimated fetal weight and/or abdominal circumference < 3^rd^ percentile or a combination of two of the three relative criteria (estimated fetal weight and/or abdominal circumference < 10^th^ percentile, slowing of the growth rate of an estimated fetal weight and/or abdominal circumference, crossing more than two quartiles on the growth percentile charts; cerebral-placental ratio < 5^th^ percentile or pulsation index in the umbilical artery greater than 95^th^ percentile) [12–14].

The criteria for inclusion in the main group were: reproductive age (18 to 45 years), singleton pregnancy, late fetal growth restriction, and patient’s consent to participate in the study. The criteria for inclusion in the comparison group were: physiological course of pregnancy, normal fetometry parameters corresponding to the gestational age, absence of uteroplacental blood flow disorders according to Doppler ultrasound, and patient’s consent to participate in the study. The exclusion criteria were: multiple pregnancy, fetal malformations and chromosomal abnormalities, severe somatic diseases and oncological diseases of the mother, refusal to participate in the study.

The diagnostic algorithm included sonography, placentography, assessment of fetal parameters, and Doppler measurement of uteroplacental blood flow by using the *Aloka SD SSD 3500* ultrasound diagnostic device (Japan) with colour Doppler mapping at 32–34 weeks and 36–38 weeks. The cerebro-placental-uterine ratio (CPUR) was calculated using the formula: ratio – pulsation index of the middle cerebral artery/pulsation index of the umbilical artery. A value below the 5^th^ percentile indicated centralization of fetal blood flow, and, according to the literature, low values were associated with a low birth weight (OR–57.0, *p* < 0.0001 – in the case of weight < 3^rd^ percentile) compared to the cerebro-placental ratio or pulsation index in the umbilical artery alone [15]. The fetal-placental coefficient (FPC): the ratio of placental weight (g) to fetal weight; an FPC value of 0.1–0.13 (as opposed to 0.15–0.2 in normal cases) may indicate placental dysfunction [15].

Postnatal macro-histomorphological assessment of the placenta was performed while taking into account the weight and dimensions of the placenta (average diameter, thickness), macroscopic features of the structure (shape, thickness, area, presence of infarctions, location of the umbilical cord insertion, nature of vascular branching, etc., placental-fetal ratio). The weight of the placenta was determined after separation of the umbilical cord and fetal membranes, washing with phosphate buffer; the diameter of the placenta was measured from the maternal surface, and the thickness was assessed by piercing with a needle in the middle and edge zones. Additionally, placental tissue samples were selected for pathohistological examination: 32 samples in the main group and 10 samples in the comparison group. Histological examination of the placenta was performed at the Educational and Scientific Laboratory of Morphological Analysis of Ivano-Frankivsk National Medical University (Prof. Zhurakivska O.Y.). The obtained material was fixed for 24 hours in 10% neutral buffered formaldehyde solution. After fixation and dehydration, the tissues were embedded in paraffin. Serial sections were obtained by using a sliding microtome and stained with hematoxylin and eosin. Light microscopy was performed by using a *Leica DM 750* microscope with ×10, ×20, and ×40 lenses and was photographed by using a digital CCD camera with a resolution of 1200×1600, whereas photos were saved in the .jpg format.

Statistical processing of the research materials was carried out by using the *STATISTICA 10* statistical analysis software package. The obtained quantitative data were first checked for normality of distribution by using Shapiro-Wilk’s W test. Since the results obtained showed their compliance with the normal Gaussian distribution, the interval (M±m) was used to describe the central tendency (typical values): arithmetic mean (Mean) ± Standard error. Accordingly, to assess the reliability of the differences in the results obtained in the main groups compared to the control group, between the data of the intact side and the side with the studied problem, a parametric t-test (Student’s criterion) was used.

Statistical processing of qualitative (categorical) data was performed by calculating the frequency of individual characteristics per 100 respondents (%), and the reliability of their differences between the comparison groups was established based on the results of χ^2^, Chi-squared test with Yate’s correction for continuity.

## Research results and discussion

The clinical characteristics of the study groups are presented in Table 1.

**Table 1:** Characteristics of the study groups, abs. number (%), n=119

Indicators	Main group, n=80	Comparison group, n=40	χ^2^; p
First-time mothers	47(58.8)	12 (30.0)	χ^2^ – 7.71; *p*<0.005
Pathological puberty, menstrual cycle disorders	17(21.3)	1(2.5)	χ^2^ – 5.96; *p*<0.01
Sexual infantilism	11(3.8)	0	χ^2^ – 4.52; *p*<0.03
Infertility	14(7.5)	0	χ^2^ – 6.32; *p*<0.01
Pregnancy after ART	10 (12.5)	0	χ^2^ – 3.94; *p*<0.05
Gestational hypertension	29(36.3)	3 (7.5)	χ^2^ – 9.85; *p*<0.001
Gestational diabetes	11(13.8)	0	χ^2^ – 4.52; *p*<0.03
Prematurity	10(12.5)	0	χ^2^ – 3.94; *p*<0.05
Excess weight	19 (23.8)	2 (5.0)	χ^2^ – 5.26; *p*<0.02
Thyroid disorders	11 (13.8)	1 (2.5)	χ^2^ – 2.60; *p*>0.05
Viral infections	14 (17.5)	1(2.5)	χ^2^ – 4.20; *p*<0.04
Placental dysfunction	28(35.03)	2 (5.0)	χ^2^ – 11.25; *p*<0.05
Oligohydramnios	26(32.5)	2 (5.0)	χ^2^ – 9.79; *p*<0.001
Operative delivery	31(38.8)	5 (12.5)	χ^2^ – 7.54; *p*<0.01
Apgar score < 7 points	30 (7.5);	2 (5.0)	χ^2^ – 12.79; *p*<0.001

The age of mothers in the main group (30.8±1.4 years) showed statistical differences (*p* < 0.05) from the average indicators in the comparison group (26.9±1.3 years). The results obtained demonstrate statistical differences in the average age of patients and parity of pregnancy. It should be noted that there is a higher incidence of FGR in cases of pathological puberty, menstrual cycle (MC) disorders and sexual infantilism, infertility and the use of assisted reproductive technology (ART), a high proportion of gestational complications (hypertensive disorders, gestational diabetes and prematurity), and somatic pathology (excess weight, viral infections). The proportion of placental dysfunction and oligohydramnios, operative delivery, and low Apgar scores was statistically significant in the main group.

Macromorphometric characteristics of the placenta and umbilical cord were assessed postnatally in both study groups. Postnatal macromorphometric features of placental tissue reflect pathology in the formation of this component of the uteroplacental complex, where, according to the results of the assessment in the main group, an abnormal shape (46–57.5%), an eccentric type of umbilical cord insertion (43–53.8%) with the main and intermediate types of vascular branching (37–46.2%) dominate.

It should be noted that the placenta weight was statistically lower (288.5±26.9 g vs. 540.8±34.8 g, *p* < 0.05), and the diameter was smaller in the main group (Figure 2). Placental-fetal ratio parameters below unity were observed in 33 cases (41.3%; χ^2^ – 15.25; *p* < 0.05).

**Figure 1:**
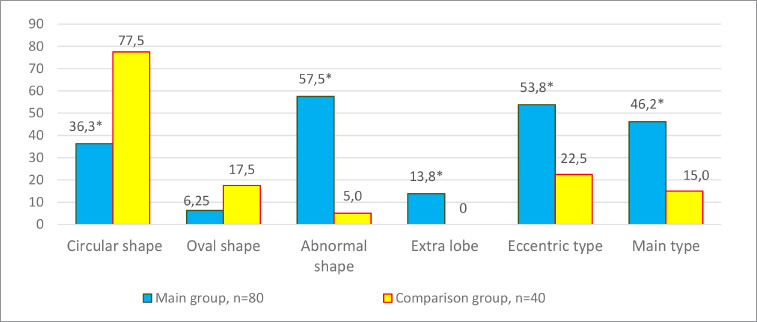
Features of postnatal macromorphology of placentas, n=120, % *Note*. * – the difference is significant compared to the comparison group data, *p* < 0.05

**Figure 2:**
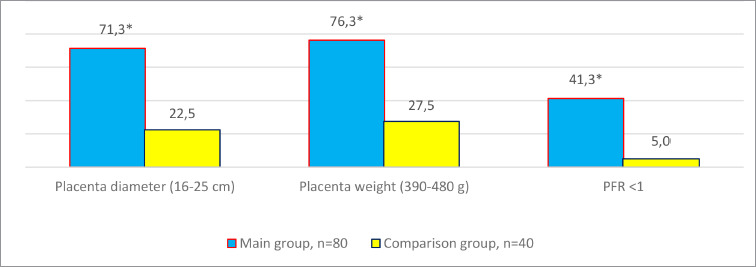
Weight and diameter of placentas in the study groups, n=120, % *Note*. * – difference is significant compared to the comparison group data, *p* < 0.05.

For histological examination, placental tissue samples were collected (32 samples in the main group and 10 samples in the comparison group). It should be noted that, according to the results of histological examination of placental tissue, pathological changes were closely related to the severity of FGR and demonstrated a combination of signs of maternal and fetal vascular malperfusion (Table 2). Among the most significant markers were: infarctions (13–40.6%; χ^2^ – 4.14; *p* < 0.04), distal villous hypoplasia (24–75.0 %; χ^2^ – 14.57; *p* < 0.03), increased syncytial nodules as a manifestation of premature placental maturation (24–75.0%; χ^2^ – 4.90; *p* < 0.03), decidual arteriopathy (13–40.6 %; χ^2^ – 4.14; *p* < 0.04). At the same time, analysis of histopathological data indicating malperfusion of fetal vessels, as well as the presence of villi and markers of intrauterine infection, was verified in more than a third of samples (13–40.6%). The obtained data complement the results of scientific research which we processed in the course of extensive scientific research [31]. Morphological markers of maternal vascular malperfusion, obtained as a result of pathohistological examination, were noted in 24 placental tissue samples (75.0%) out of 32 obtained postnatally in the main group (*p* < 0.001).

**Table 2:** Micromorphological features of placental tissue, n=42, abs. number (%)

Microscopic patterns of placental dysfunction		Main group, n=32	Comparison group, n=10	χ^2^, p
Maternal vascular malperfusion	decidual arteriopathy	13 (40.6)	0	χ^2^ – 4.14; *p*<0.04
	agglutinated villi	14 (43.8)	0	χ^2^ – 4.74; *p*<0.03
	enlargement of syncytial nodules	24 (75.0)	3(30.0)	χ^2^ – 4.90; *p*<0.03
	placental site infarction	13 (40.6)	0	χ^2^ – 4.14; *p*<0.04
	anchoring villi infarction	12(37.5)	0	χ^2^ – 3.57; *p*>0.05
	hypoplasia of the distal part of the villi	24 (75.0)	0	χ^2^ – 14.57; *p*<0.03
Fetal vascular malperfusion	avascular villi	23 (71.9)	3(30.0)	χ^2^ – 4.03; *p*<0.04
	stromal-vascular karyorrhexis of villi	1(3.1)	0	χ^2^ – 0.39; *p*>0.05
	obliteration of stem vessel arteries	13(40.6)	0	χ^2^ – 4.14; *p*<0.04
	thrombi in the chorionic plate or stem villi	3(9.4)	0	χ^2^ – 0.09; *p*>0.05
	obstructive lesions of the umbilical cord	7(21.9)	0	χ^2^ – 1.29; *p*>0.05
	delayed villi maturation	14(43.8)	0	χ^2^ – 4.74; *p*<0.03
	villous hyalinosis	13(40.6)	0	χ^2^ – 4.14; *p*<0.04
	vascular ectasia	11(34.4)	0	χ^2^ – 3.05; *p*>0.05
	chorionic villi edema	15(46.9)	0	χ^2^ – 5.39; *p*<0.02

**Figure 3:**
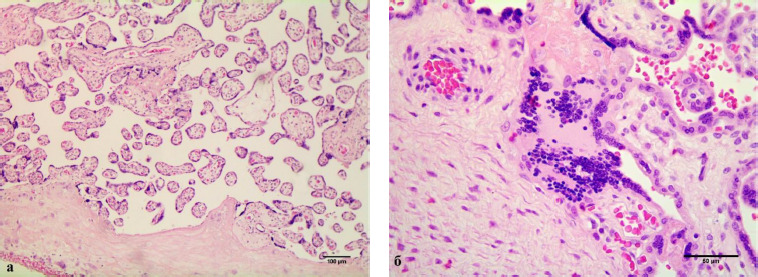
Pathomorphological signs of placental dysfunction in a patient with FGR (patient A, diagnosis: pregnancy I, 36 weeks of pregnancy, fetal growth restriction < 3^rd^ percentile, late form). Hypoplasia of terminal chorionic villi with an increase in syncytial nodules (a). Lymphoplasmacytic villitis, vascularization of chorionic anchoring villi and delayed maturation (b). Staining: hematoxylin and eosin. Magnification: a) x100, b) x400

In cases of maternal vascular malperfusion, villous hypoplasia was diagnosed. The villi become elongated and thinner, and the distance between the terminal (tertiary, distal) villi increases, leading to an increase in the intervillous space (Fig. 1 a). This is usually associated with a placenta that is small for gestational age. Another change characteristic of placentas with maternal vascular malperfusion is accelerated villous maturation, characterized by marked hyperplasia of syncytial nodules (Fig. 3a). Increased nuclear agglutination and basophilia of multinucleated cells on terminal villi, i.e., hyperplasia of syncytial nodules, is often observed in combination with terminal villous hypoplasia and is usually associated with maternal hypertension or uteroplacental insufficiency. Accelerated villous maturation is accompanied by alternating areas of villous agglutination and syncytial nodules with areas of reduced density and branching of terminal villi. Quite often, such placentas have large fused syncytial nodes and entire foci of intervillous fibrinoid in the area adjacent to the anchoring villus.

Such pathologies as villous infarction with clearly defined contours, collapse of the intervillous space, intervillous fibrosis, caryorectic remnants, neutrophilic and histomonocytic infiltration of villi and intervillous space, loss of basophilia of trophoblasts and villi stroma are not common. Decidual arteriopathy or acute atherosis is a pathognomonic sign of maternal vascular malperfusion. It is a more severe stage of fibrinoid necrosis, involving the accumulation of foamy macrophages embedded in the vessel wall, which are best seen in the arteries in placental bed biopsies, but can also spread to the decidual arteries. Less common cases are the following: decidual necrosis, pseudocysts of the chorionic lymphatic artery, islands of fibrin with extravillous trophoblasts; and a significant increase in intervillous fibrin.

**Figure 4:**
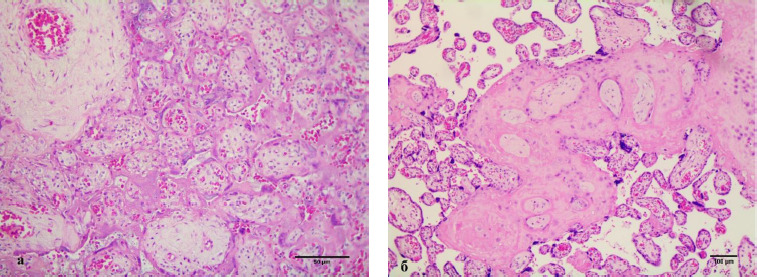
Histopathological signs of fetal vascular malperfusion (patient B, pregnancy II, 37 weeks of pregnancy. Fetal growth restriction < 3 percentile, late form). Hyalinosis and edema of villi with narrowing and complete disappearance of the intervillous space in some areas, vascularization of terminal villi (a). Obliteration of the vessels of the anchoring villi and expansion of the intervillous space with an increase in syncytial nodules on the terminal villi (b). Staining: hematoxylin-eosin (a–b). Magnification: a) x400, b) x100

Assessment of histological features of the placenta in 3 of 32 samples (9.4%) revealed high-grade fetal vascular malperfusion at various stages of development. Stem vessels of 2–4 orders were diagnosed with thrombus in initial recanalization and partial fibrinoid organization. The surrounding villi were vascularized, immature with hyalinized syncytiotrophoblast and completely absent trophoblast with varying degrees of intravillous stromal fibrosis (Fig. 4a) due to prolonged ischemia. In 13 samples (40.6%) of the placentas studied, the obliteration of the 2^nd^ and 3^rd^ order spiral arteries in the anchoring villi was found (Fig. 4 b).

Quite rarely (1–3.1%), in the fetal part of the placenta with FGR, stromal-vascular caryorrhexis of villi was diagnosed. It was characterized by the presence of caryorrhexis of embryonic cells (nuclear erythrocytes, leukocytes, endothelial cells and/or stromal cells) in 3–4 terminal villi with preservation of the trophoblast.

In one third of the cases researched, a delayed villous maturation was observed. It was characterized by the presence of more than 10 villi with centrally located capillaries, a continuous layer of cytotrophoblasts, and a reduced number of syncytial-capillary membranes, which replicates the histological picture of early pregnancy (up to 28 weeks of gestation), but diagnosed after 36 weeks of pregnancy.

In 11 samples (34.4%) out of the researched cases, vascular ectasia was detected as both 1^st^ and 2^nd^ order vessels in the anchoring vessels of the villi and varicose veins or arteries in the umbilical cord, which were combined in 60% of cases.

Chronic inflammatory processes of the placenta were extremely rare in patients with FGR (3–9.4%). In three cases (9.4%), we diagnosed villitis of an unknown etiology, characterized by a placenta that was too large for the gestational age and damage to the anchoring villi. Sections of the parenchyma showed somewhat immature anchoring villi, diffusely affected by lymphoplasmacytic infiltrate (Fig. 3 b) and the development of plasmacytic deciduitis.

## Discussion

The etiopathology of FGR caused by abnormal development of uteroplacental blood flow and its impact on the development and structure of the placenta has been studied for over five decades. Modern examination methods (ultrasound, 3-dimensional Doppler imaging, etc.) allow for the study of both umbilical-placental and uteroplacental blood flow starting from the first trimester of pregnancy and are widely used for screening placental complications of pregnancy, such as preeclampsia and management of a fetus with primary or secondary FGR [9, 16]. Prenatal diagnosis of placental and umbilical cord abnormalities allows for specialized prenatal care, fetal monitoring and birth planning, which can lead to better maternal and perinatal outcomes [9].

For better diagnosis of fetoplacental circulation disorders, the *Amsterdam International Consensus Group of Placental Pathologists* introduced a new term ‘fetal vascular malperfusion’ in 2015 and developed criteria for assessing developmental pathology and placental dysfunction [10, 17–18]. According to their recommendations, all types of placental pathology should be classified into 3 groups: maternal vascular malperfusion, fetal vascular malperfusion, and chronic inflammation. Such phenotypes and risks of recurrence of FGR associated with specific types of placental pathology are also recommended by the standards of medical care for FGR approved by the order of the Ministry of Health of Ukraine in 2023 [4].

Research results indicate that placentas with early FGR may have pathological lesions of two categories: maternal vascular malperfusion (infarction, villous hypoplasia, increased syncytial nodules, and increased nuclear blood flow in fetal vessels) and chronic inflammatory processes (chorioamnionitis, villitis, and acute funisitis) [19].

According to our research, pathological placental lesions in FGR occurred in two ways – maternal vascular malperfusion and fetal vascular malperfusion. Meanwhile, chronic inflammatory lesions of the placenta were extremely rare. In this case, the segmental type of fetal vascular malperfusion prevailed, indicating thrombotic occlusion of chorionic or stem villous vessels, or obliteration of stem vessels – although the distribution of lesions is segmental, a thrombus or obstruction is likely to lead to complete obstruction of the villi downstream, which may be a prerequisite for miscarriage, premature birth and fetal distress during pregnancy.

A recent population-based study linked global fetal vascular malperfusion to neonatal encephalopathy [20]. Pathomorphological features of fetal vascular malperfusion in these cases included: small foci of ischemic villi; vascular ectasia and intramural fibrin deposition, which were associated with cord entanglement, intrauterine fetal death, and fetal arrhythmias [21–22].

Such placental changes were associated with developmental delay, oligohydramnios, chronic fetal monitoring abnormalities, neonatal coagulopathies (thrombocytopenia, thromboembolic disease) and fetal cardiac anomalies [10, 21].

Moreover, it has been shown that high levels of fetal vascular malperfusion (fetal thrombotic vasculopathy) are closely correlated with CNS abnormalities such as cerebral palsy, neonatal encephalopathy, perinatal stroke, and intracranial hemorrhage [10, 23–24].

Less well-established associations with fetal vascular malperfusion include maternal diabetes and a variety of fetal/neonatal abnormalities, including necrotizing enterocolitis, congenital leukemia, neonatal autoimmune thrombocytopenia, and non-cardiac congenital malformations [25].

It is worth noting a number of literature reports where an important factor determining the severity of clinical manifestations of FGR is damage to the vascular bed of the placental villi at all levels, where obliterative angiopathy of resistance villi affects the degree of capillarization of terminal villi and a decrease in the number of syncytiocapillary membranes and can serve as a criterion for the severity of the pathology of perfusion of the uteroplacental blood flow [26].

One of the morphological concepts of placentology, such as ‘obliterative angiopathy’ of the arterioles of the resistance villi of the placenta, was first described by the German pathologist V. Becker (1981); however, scientific positions on hypertrophy of the muscular layer and a decrease in the diameter of the lumen of the arterioles in the resistance villi are quite controversial, and are most often found in women smokers, with severe preeclampsia [18, 27–31], and reflect, in the opinion of the authors, complex hemodynamic placental-fetal-umbilical disorders. In the literature, there are ambiguous explanations and descriptions of placental tissue with morphogenesis of the obliterative process in the arterioles [26, 32].

There are opinions about the deficiency and vasodilation failure of umbilical vessels and arterioles of resistance villi [18–19, 30]; there are no studies on the morphometric characteristics of the obliteration process in arterioles as a marker of regulatory mechanisms that cause hypertrophy of the muscle layer and a decrease in the lumen of arterioles [19]. The scientific statements obtained according to the literature search were confirmed by a certain morphological evidentiary substrate as a result of our scientific research (Fig. 4b).

## Conclusions

Postnatal macromorphometric characteristics of placental tissue reflect pathological features of its formation, where an abnormal shape dominates (57.5%), followed by eccentric type of umbilical cord insertion (53.8%) with the main and intermediate type of vascular branching (46.2%), a statistically lower placental mass and its diameter. According to our studies, in the case of late form of fetal growth restriction, pathological lesions of the placenta, as a rule, occur in two ways: with the development of maternal and fetal vascular malperfusion. Pathomorphological criteria of maternal vascular malperfusion in placentas of parturient women with fetal growth restriction were statistically significantly more often noted: infarctions, distal villus hypoplasia, decidual arteriopathy and dysmaturation of chorionic villi. Fetal vascular malperfusion was characterized by obliteration and thrombosis of the stem vessels of anchoring and intermediate villi, avascularization, hyalinosis of villi and less often by delay in their maturation with the development of stromal-vascular karyorrhexis. In our study, in placental tissue samples in the case of fetal growth restriction, both 1^st^ and 2^nd^ types of fetal vascular malperfusion were detected, however, its segmental type dominated.
